# Genome-wide mapping of EBV-induced genomic variations identifies the role of MUC19 in EBV latency

**DOI:** 10.1128/mbio.02055-25

**Published:** 2025-09-25

**Authors:** Jingwen Yu, Yaohao Wang, Qirong Liu, Xiaohui Zhou, Erle S. Robertson, Yonggang Pei

**Affiliations:** 1 School of Public Health and Emergency Management, Southern University of Science and Technology 638536, Shenzhen, Guangdong, China; 2 School of Medicine, Southern University of Science and Technology 255310, Shenzhen, Guangdong, China; 3 The Tumor Virology Program, Department of Otorhinolaryngology-Head and Neck Surgery, Perelman School of Medicine at the University of Pennsylvania 14640 , Philadelphia, Pennsylvania, USA; 4 Department of Microbiology, Perelman School of Medicine at the University of Pennsylvania 14640 , Philadelphia, Pennsylvania, USA; The University of North Carolina at Chapel Hill, Chapel Hill, North Carolina, USA

**Keywords:** EBV, CNV, EBNA1, MUC19, tandem repeat, mTOR pathway, latency

## Abstract

Epstein-Barr virus (EBV) infects over 95% of the world’s population and is tightly associated with multiple human malignant diseases. As the first discovered human oncovirus, EBV is known to induce genomic instability by promoting various types of genomic modifications in host chromosomes. However, the mechanisms through which EBV interacts with the host genome and regulates cellular gene expression in genomic modifications are not yet fully elucidated. In this study, we conducted primary EBV infection in B cells and performed the analyses of copy number variants using whole genome sequencing. The results showed genomic regions susceptible to EBV-induced mutations and unveiled MUC19 to be a critical host factor in EBV latency, which was distinctively activated transcriptionally upon EBV infection. Finally, we identified the intrinsic tandem repeats in MUC19 to be its functional domain, which promotes cell survival and cell cycle through activating mechanistic target of rapamycin (mTOR) signaling in EBV-positive cells. Further results indicate that EBV nuclear antigen 1 binds to the promoter of the MUC19 gene and enhances its expression. In conclusion, these results provide novel insights into the roles of MUC19 in EBV latency, highlighting its potential as a promising therapeutic target for the treatment of EBV-associated lymphomas.

**IMPORTANCE:**

Genomic instability is a hallmark of cancer. EBV contributes to host genomic instability after primary infection. This study maps the EBV-induced genomic variations using deep whole genome sequencing and identifies the critical factor MUC19, which is one of the most understudied genes, with a genomic sequence exceeding 177 kbp that encodes a protein over 800 kD. In this study, we revealed that EBV induced the duplicated copy number variants of the MUC19 gene and enhanced its expression, which further promotes cell survival and cell cycle via mTOR signaling. Overall, this study maps the genomic perturbations induced by EBV primary infection and offers new insights into the critical role of MUC19 in EBV latency.

## INTRODUCTION

Epstein-Barr virus (EBV), also known as human herpesvirus 4 (HHV-4), infects over 95% of the global population, making it one of the exceptionally successful pathogens. EBV infection is closely associated with a variety of human diseases, including Burkitt’s lymphoma (BL), Hodgkin’s lymphoma, infectious mononucleosis, nasopharyngeal carcinoma (NPC), gastric cancer, and multiple sclerosis (1, 2). Following initial infection in the host’s oral epithelial cells, EBV subsequently releases progeny virus particles to infect host B lymphocytes, establishing latent infection in the form of a circular extrachromosomal episome (3).

Genomic instability is a hallmark of cancer and is characterized by genomic mutations (4). These aberrant mutations have the potential to induce the oncogenic phenotype and trigger tumorigenesis (5, 6). EBV is reported to promote genomic instability in B lymphocytes (7). For instance, the latent EBV episome causes genomic instability by frequently interacting with the host genome (8). The advancement of high-throughput sequencing methods facilitates the identification of distinct chromosome modifications with higher precision (9, 10). For instance, the genome-wide association study on single-nucleotide mutations from 681 clinical non-Hodgkin lymphoma (NHL) patients and 749 controls identified key mutations in diffuse large B-cell lymphoma (DLBCL) within LOC283177 and confirmed mutations associated with chronic lymphocytic leukemia at chromosomes 13q14, 11q22-23, 14q32, and 22q11.22 (11). Compared to small genomic variations on a single-nucleotide level, structural variations (SVs) on a copy number level bear the potential to further identify the chromosomal variations induced by viral antigens. Copy number variants (CNVs) typically refer to genomic fragment variations over 1 kb in the human genome and are known to contribute to multiple malignant cancers, including lymphomas (11–14). Therefore, studying the phenotypic impact of CNVs in EBV-positive cells has the potential to reveal novel variations and deepen our understanding of EBV-associated oncogenesis.

Previous studies in at least 12 lymphoblastoid cell lines (LCLs) showed that EBV infection induces chromosome modifications across more than 70 chromosomal bands (*P* < 0.05) with shared integration at 1p31, 1q43, 2p22, 3q28, 4q13, 5p14, 5q12, and 11p15 (15). In Burkitt’s lymphoma, EBV integration leads to large genomic deletions (dels) in the viral genome, including regions that contain the *LMP* and *EBER* genes. Meanwhile, the integrated viral genome disrupts chromosome stability within infected cells, leading to translocations and deletions on chromosomes 11 and 19 (8). Spectral karyotyping using the primary EBV infection model identified widespread chromatin and telomere abnormalities on the host genome that occurred 4 weeks after post-infection (16). Moreover, EBV-targeting genomic perturbations contribute to oncogenesis via disrupting genes, activating oncogenes, and increasing host genomic instability (17). Collectively, these studies confirm that EBV is capable of interacting with the host genome in B lymphocytes and inducing genomic variations to reshape the B-cell genomic landscape. However, the mechanisms by which EBV induces these mutations and their oncogenic pathways remain largely unexplored.

EBV proteins such as BNRF1 and EBV nuclear antigen 1 (EBNA1) are shown to mediate host chromosomal structural variations. BNRF1 induces SVs and is essential for the replication and maintenance of EBV latent infection (7). EBNA1 attaches EBV episomes to host genome through OriP during latency by forming a replication-dependent cross-structure with host DNA (18). A recent study revealed that the aggregation of EBNA1 around the host chromosome at 11q23 causes double-strand breakage in EBV-positive B lymphocytes, potentially inducing genomic mutation (19). The following results showed that EBV episomes attach to the human genome through clusters of 18 bp palindromic repeats homologous to the sequence on host chromosome 11q23, disrupting chromosomal stability (19). This study provides novel insights into EBV-induced mutations on chromosome 11q23, while EBV’s ability to induce perturbations across the human genome suggests the involvement of additional mechanisms.

Here, we conducted an integrated analysis of EBV-associated genomic variations across different B-cell lines using whole genome sequencing (WGS) and highlighted the specific genomic CNVs induced by EBV primary infection. We discovered that EBV infection triggers the copy number duplication (dup) of the MUC19 repeat region and enhances MUC19 expression to promote cell survival and cell cycle, suggesting its oncogenic role. These findings provide new insights into EBV-induced genomic variations and highlight MUC19 as a novel target for anti-EBV therapeutics.

## RESULTS

### EBV can induce specific CNVs in B cells

**Fig 1:**
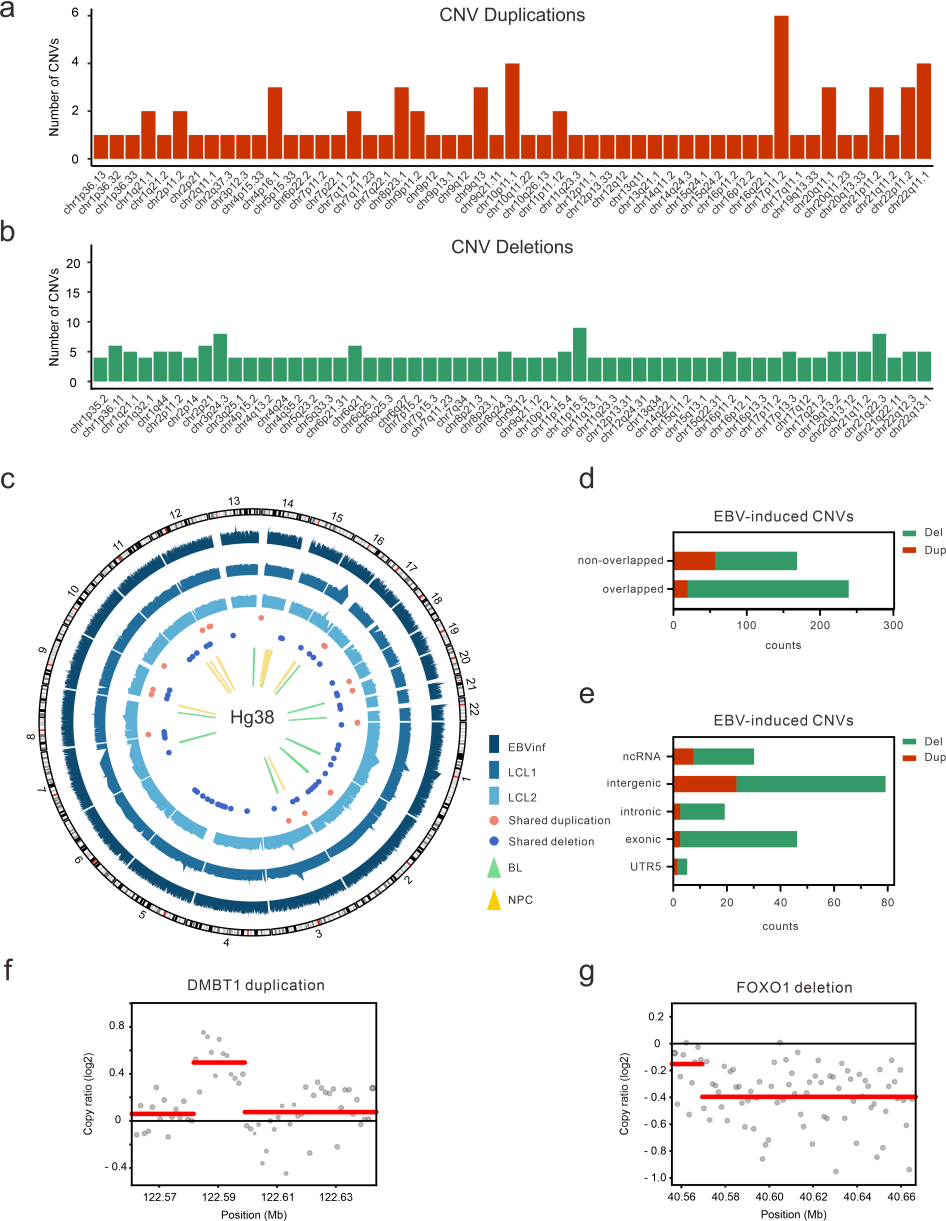
EBV induces specific CNVs in B cells. (**a and b**) CNVkit was used to identify CNV duplications (**a**) and deletions (**b**) from WGS data in EBV primary infection, and the CNV regions were mapped to the Hg38 cytoBand annotation. All CNVs in autosomes are shown. (**c**) WGS was performed on the genomic DNA from EBV-positive cells (EBVinf, LCL1, and LCL2), and then CNV analyses were conducted using the CNVkit. EBVinf refers to the B cells collected after 2 weeks of EBV infection. Dots denote shared duplications (red) and deletions (blue) of all three EBV-positive cell samples. CNVs for BL and NPC are obtained from studies describing EBV-specific chromosomal fragile sites (20–22). (**d**) EBV-induced CNVs overlapped with the topologically associating domains in EBV-positive GM12878 cells. EBV-induced CNVs are defined as the CNVs shared between EBVinf and LCLs that are not observed in uninfected primary B cells. (**e**) EBV-induced CNVs were mapped to the Hg38 refGene annotation to reveal the characteristics of these CNV regions. (**f and g**) The scatter plots display the mutated oncogenes as a result of EBV infection. *DMBT1* is shown to be duplicated (**f**), and *FOXO1* contains EBV-induced segmental deletion (**g**). The red line indicates the average copy ratio per segment. A copy ratio (log2) exhibits the log2 transformed value of CNV in tumors by reflecting deviations from diploid coverage.

To investigate the EBV-induced genomic changes, we performed EBV primary infection in B cells and collected the infected (EBVinf) or uninfected B cells (total-B) for WGS. Then, CNV analysis revealed 53 distinct dups and 469 dels, highlighting EBV-induced genomic instability (Fig. 1a and b). Frequent CNV duplications were observed at chr17p11.2 (six dups), chr10p11.1 (four dups), and chr22q11.1 (four dups), while frequent deletions were detected at chr11p15.5 (nine dels), chr21q22.3 (eight dels), and chr3p24.3 (eight dels). We next sequenced two LCLs and identified shared CNVs of these three EBV-positive samples (Fig. 1c). Interestingly, the shared CNVs are found to overlap with known EBV-specific chromosomal fragile regions in BL and NPC (20–22), indicating the potential to study EBV-induced genomic variations in EBV-mediated diseases (Fig. 1c). To examine the characteristics of EBV-mediated CNVs, we mapped the CNV regions to the high-throughput chromosome conformation capture (Hi-C) results from EBV-positive GM12878 cells, which defined the topologically associating domains (TADs) with active transcription (23). This analysis revealed that the majority of EBV-induced CNVs overlap with TADs, with most deletions occurring within these regions (Fig. 1d), demonstrating that EBV infection can induce genomic variations and modulate TAD architecture. Additionally, the shared segmental duplications are generally within non-coding and intergenic regions (Fig. 1e), which suggests the potential functions of non-coding regions in EBV-induced oncogenesis.

The identified CNVs included known oncogenes, such as *DMBT1* and *FOXO1* (Fig. 1f and g). Rearrangements within *DMBT1* are frequently discovered in multiple tumors (24, 25), and mutated *FOXO1* is a known marker for decreased overall survival in DLBCL patients associated with failure to achieve event-free survival at 24 months (EFS24) after diagnosis (14, 26). Primary infection induced copy number duplication within *DMBT1* and deletion in *FOXO1*, highlighting their essential functions during EBV infection. Besides CNV analyses, we performed the single-nucleotide polymorphism (SNP) analyses on EBV-infected primary B cells, which revealed 4,369,022 EBV-induced SNP sites (Fig. S1a). After normalizing the SNP counts based on the lengths of their CNVs, we further defined the 100 most genomically unstable CNV regions, which overlap with 233 genes. These selected genes were involved in specific virus infection and host defense (Fig. S1b). Therefore, we identified EBV-induced CNVs in host chromosomes from EBV primary infection using WGS analyses, which may play a crucial role in EBV-mediated diseases.

### CNV duplication of MUC19 is associated with its enhanced expression

**Fig 2:**
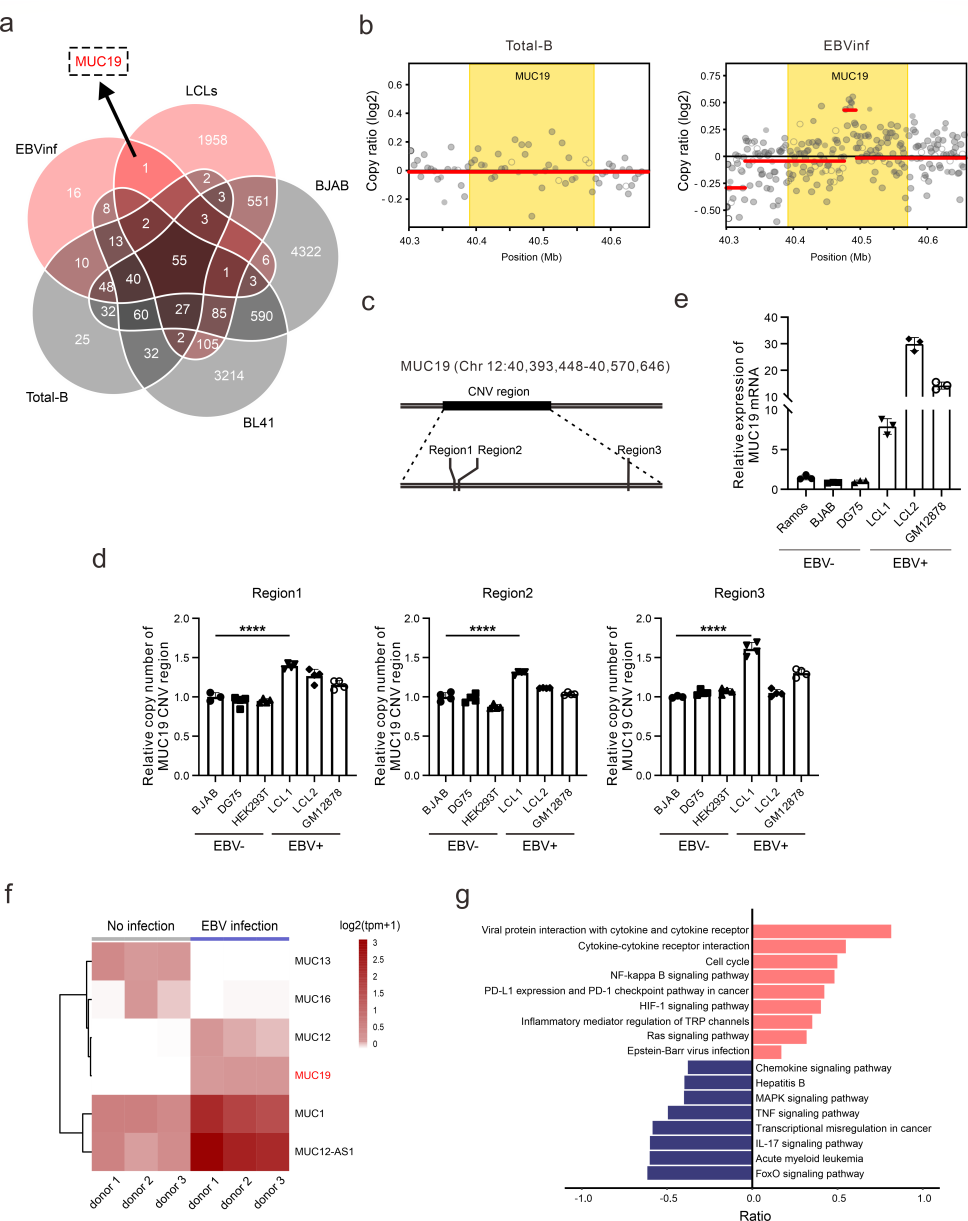
CNV duplication of MUC19 is associated with its enhanced expression. (**a**) The comparative analysis identifies shared CNV duplications within *MUC19* among EBV-positive cell lines. BL41 and BJAB, EBV-negative Burkitt’s lymphoma cells; EBVinf, the B cells after 2 weeks of EBV infection; LCL, the combination of EBV-positive LCL1 and LCL2 cells; total-B, the primary B cells without EBV infection, which share a similar background with the EBVinf cells. (**b**) CNV analyses show that *MUC19* is duplicated by EBV primary infection when comparing primary B cells (total-B) to EBV-infected B cells (EBVinf). (**c**) A schematic of designed primers to validate the identified CNVs in *MUC19*. (**d**) The copy number of identified CNVs within *MUC19* was determined in EBV-positive or EBV-negative cell lines. ****, *P* < 0.0001. (**e**) Real-time PCR was conducted to detect MUC19 mRNA expression in EBV-positive or EBV-negative cell lines. (**f**) RNA-seq analysis shows that multiple mucin factors were differentially expressed during EBV primary infection. The samples on days 0 and 14 were obtained from the GEO data set GSE125974, with a *P* value threshold of 0.05. (**g**) Kyoto Encyclopedia of Genes and Genomes analysis on the differentially expressed genes (DEGs) reveals the important cellular signaling pathways regulated by EBV primary infection. An absolute fold change of 5 was used as a threshold for differentially expressed genes. MAPK, mitogen-activated protein kinase.

To further identify key genes associated with the EBV-specific CNVs, we performed a comparative analysis between EBV-positive (EBVinf, LCL1, and LCL2) and EBV-negative (BL41, BJAB, and total-B) samples. The results revealed that only one gene, *MUC19*, was specifically duplicated by EBV infection (Fig. 2a), while seven other genes were found to be partially deleted (Fig. S2a). Moreover, our results suggest that EBV infection in B cells introduced a 17,368 bp duplication located within the middle of the MUC19 gene (Fig. 2b). Additionally, this duplication was determined by targeting specific regions of the potential CNV duplication across several EBV-associated cell lines. The results indicated that EBV-positive cells exhibit a higher copy number of this region compared to EBV-negative cells (Fig. 2c and d). We then propose that such duplication may lead to upregulation of MUC19 in EBV-positive B cells, and the following results showed that *MUC19* expression is significantly higher in EBV-positive cell lines (LCL1, LCL2, and GM12878) compared to the EBV-negative cell lines (Ramos, BJAB, and DG75) (Fig. 2e), which suggests that MUC19 may be specifically upregulated in EBV latency.

Duplicated genes within CNV regions are shown to regulate pathogenesis by overexpression through the dosage effect (27). To further explore the expression changes of CNV-associated genes during EBV primary infection, we analyzed the transcriptional profile of EBV primary infection in B cells (Fig. S2b) (28). The results showed that MUC19 expression was upregulated in EBV-infected B cells 14 days post-infection (Fig. 2f). Enrichment analysis of differentially regulated genes from transcriptome further demonstrated the critical signaling pathways during EBV infection, including cell cycle, NF-κB signaling, and tumor necrosis factor (TNF) signaling (Fig. 2g), which are well-established pathways associated with EBV infection and its ability to drive proliferation (29). Additionally, several EBV-associated transcription factors also colocalize at the LRRK2/MUC19 region, highlighting its significance in EBV-mediated pathogenesis (Fig. S2c). Therefore, these findings indicate that EBV activates MUC19 expression upon infection, suggesting a contributing role of MUC19 in EBV latency.

### MUC19 promotes cell cycle in EBV-positive B cells

**Fig 3:**
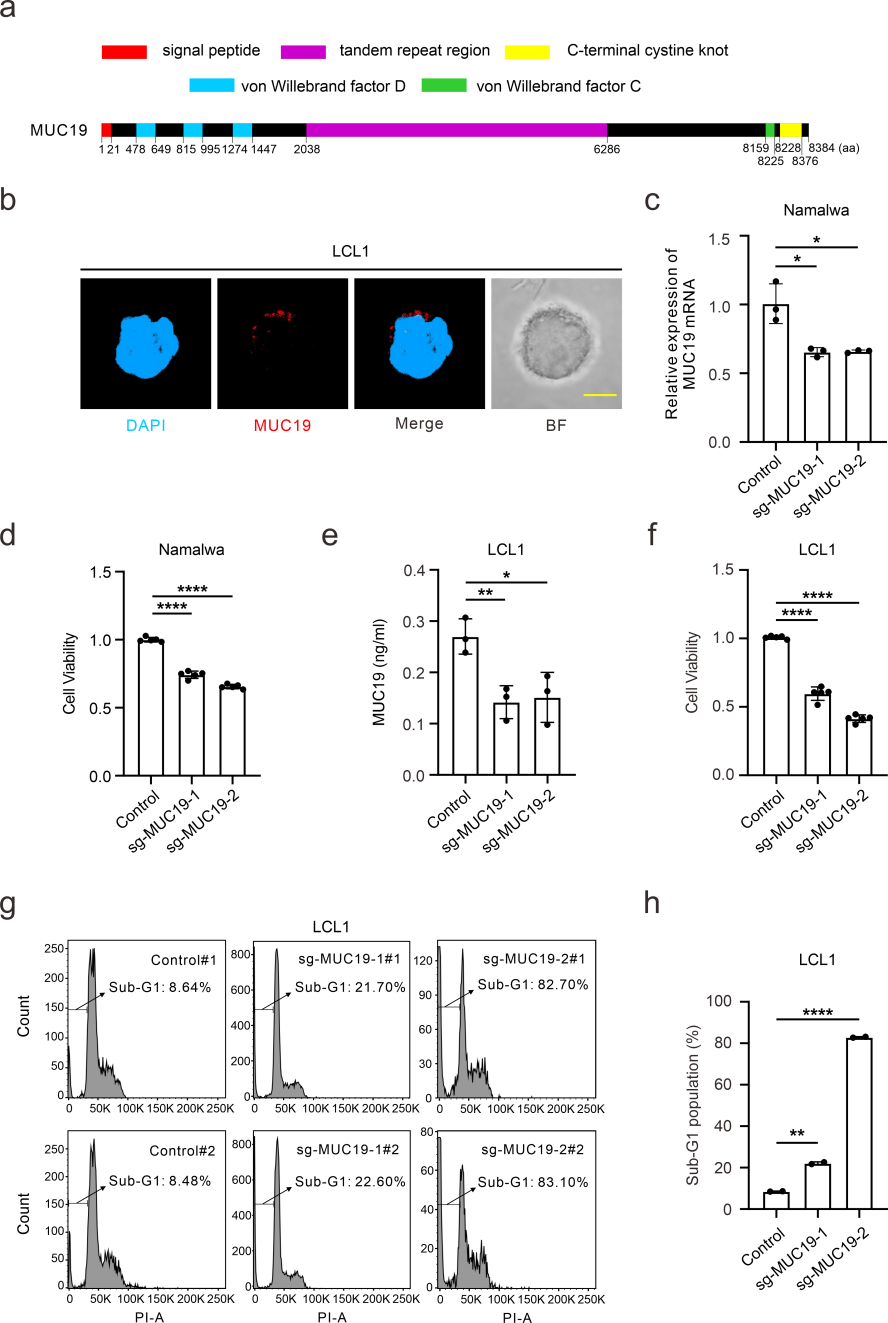
MUC19 promotes cell cycle in EBV-positive B cells. (**a**) A schematic diagram illustrates the structure of MUC19 protein. The key features are based on the annotations available for Uniprot entry Q7Z5P9. (**b**) The endogenous MUC19 expression was detected using a specific primary antibody followed by the secondary staining with Alexa Fluor 594 (red) in EBV-positive LCL1 cells. The nuclei were stained with 4′,6-diamidino-2-phenylindole (DAPI; blue). BF, bright field. Scale bars, 5 µm. (**c**) The knockdown efficiency of MUC19 mRNA expression with quantitative PCR was determined in Namalwa cells using the CRISPRi system. (**d**) CCK-8 assay shows cell viability after MUC19 knockdown in Namalwa cells. (**e**) The efficiency of MUC19 knockout using the CRISPR/Cas9 system in LCL1 cells was assessed using enzyme-linked immunosorbent assay. (**f**) CCK-8 assay shows the cell viability after MUC19 downregulation in LCL1 cells. (**g and h**) Flow cytometry was utilized to determine cell cycle and apoptosis induced by MUC19 downregulation in LCL1 cells, which were fixed with 70% ethanol and stained with propidium iodide (PI). *, *P* < 0.05; **, *P* < 0.01; ***, *P* < 0.001; ****, *P* < 0.0001.

Human mucin gene *MUC19* (chr12: 40,393,394–40,570,832) is over 177 kbp in length and encodes a protein of 8,384 amino acids with mucin-like threonine/serine-rich repeats (30). MUC19 has three von Willebrand factor D domains near its N-terminus, and a von Willebrand factor C (VWC) domain followed by a C-terminal cystine-knot (CTCK) domain (Fig. 3a). As a member of the mucin family, MUC19 shares close homology with MUC2, MUC6, MUC7, MUC5A, and MUC5B (Fig. S3a). Previous studies showed MUC19 secretion in glandular tissues and epithelial cells, including major salivary glands (31), lacrimal glands (32), middle ear epithelium (33), and airway tissues (34). However, our results indicate that MUC19 localizes in the cytoplasm (Fig. 3b) and shows no detectable secretion in EBV-associated cell lines (Fig. S3b), demonstrating that MUC19 primarily functions within the cytoplasm in these cells.

To explore the potential oncogenic role for MUC19 in B lymphoma cells, we generated MUC19 knockdown cells using CRISPRi and CRISPR/Cas9 systems (35, 36), both of which inhibit MUC19 expression (Fig. 3c and e). Then, the following assays showed that MUC19 downregulation significantly reduced cell viability (Fig. 3d and f). This abrogated viability suggests an important role for MUC19 in maintaining cell growth and survival. Furthermore, MUC19 suppression in LCL1 caused cell cycle arrest and triggered apoptosis (Fig. 3g). The indicated cells are marked by a significantly elevated proportion of apoptotic cells (Sub-G1) (Fig. 3h). These findings were consistent with those observed in Namalwa cells using the CRISPRi system (Fig. S3c and d). In summary, we characterized the localization of MUC19 in EBV-positive lymphoma cells and identified its role in promoting cell survival and cell cycle using distinct CRISPR systems.

### MUC19 can activate the mechanistic target of rapamycin pathway through its repeat region

**Fig 4:**
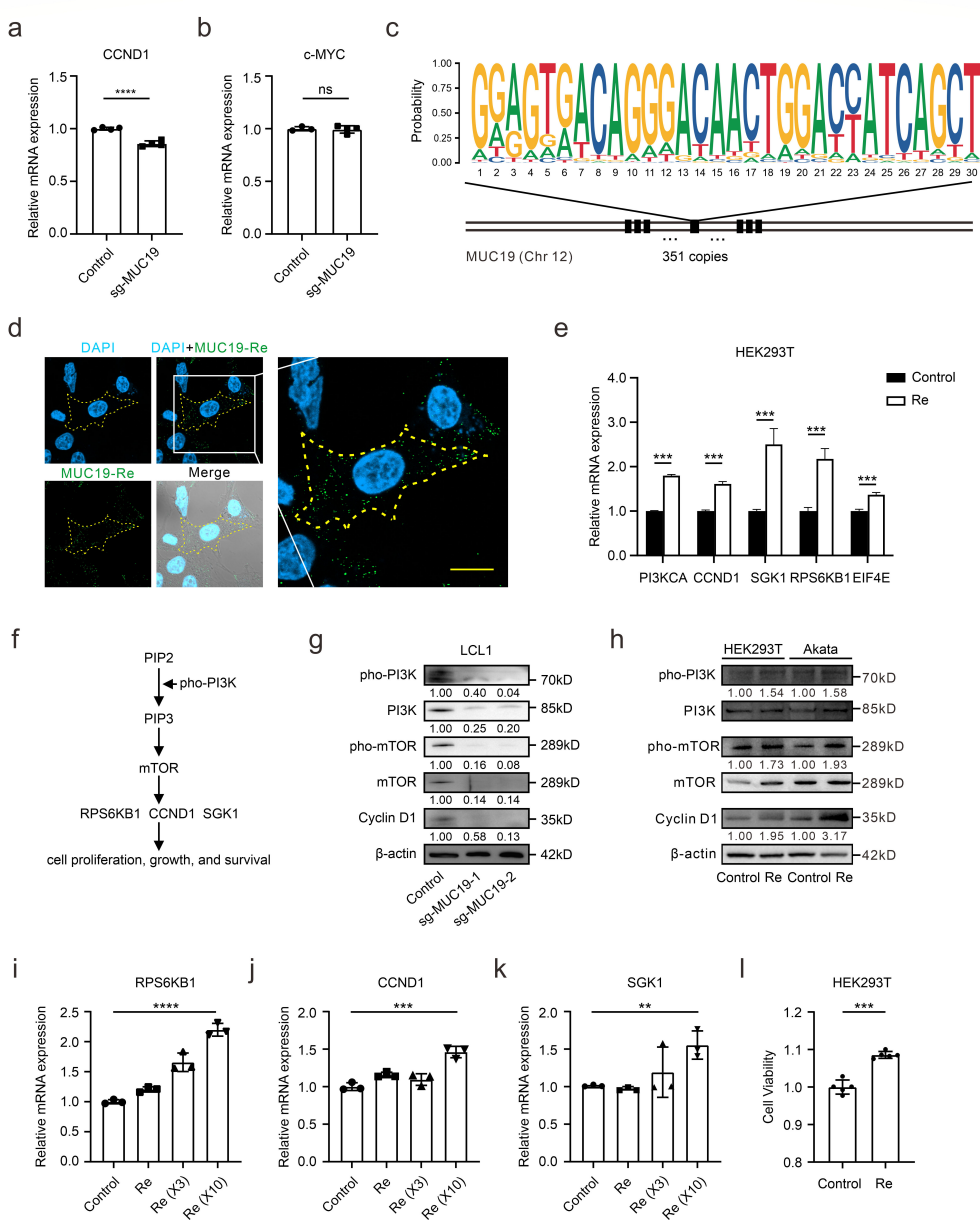
MUC19 can activate the mechanistic target of rapamycin (mTOR) pathway through its repeat region. (**a and b**) The expression of CCND1 (**a**) or c-MYC (**b**) in Daudi cells was detected after MUC19 suppression. The sgRNA used here corresponds to the sgMUC19-1 in LCL1, delivered with the CRISPR/Cas9 system. (**c**) The motif analysis tool FIMO was employed to search for the 30 bp basic repeat unit in MUC19 DNA (as shown), which encodes the tandem repeat “GVTGTTGPSA.” FIMO identified a total of 351 copies of the 30 bp repeat in *MUC19*, with 338 located within the duplicated region. A *P* value threshold of 0.00002 was applied. (**d**) The localization of the basic repeat unit (with Myc tag) was determined in HEK293T cells. Re was stained with Alexa Fluor 488 (green), and cell nuclei were stained using DAPI (blue). The yellow dotted line denotes the cell membrane of a single HEK293T cell. Re, the 30 bp basic repeat unit in MUC19 DNA. Scale bars, 10 µm. (**e**) HEK293T cells were transfected with Re plasmid or not, and then qPCR was performed to determine mRNA expression of the indicated targets. (**f**) The schematic illustrates the mTOR-related signaling pathway. (**g**) The expression of key factors in the mTOR signaling pathway was examined after MUC19 downregulation in LCL1 cells. The antibody for pho-PI3K targets P85α/β/P55γ, whereas the antibody for PI3K targets P85α. (**h**) The expression of indicated cellular factors was determined following Re overexpression in HEK293T and Akata cells at 48 h post-transfection. (**i–k**) The expression of *RPS6KB1* (**i**), *CCND1* (**j**), or *SGK1* (**k**) was determined 48 h following the expression of multiple copies of Re. Re (×3), 3 copies of the 30 bp repeat; Re (×10), 10 copies of the 30 bp repeat. ***, *P* < 0.001; ****, *P* < 0.0001. (**l**) CCK-8 was utilized to assess cell viability after Re overexpression in HEK293T cells.

Previous studies have indicated that the C-terminus of MUC1 triggers Wnt/β-catenin pathway and increases SNAIL transcription to activate epithelial-mesenchymal transition (EMT) in cancer cells (37). To reveal the mechanisms underlying MUC19’s role in promoting cell cycle, we determine how MUC19’s C-terminal domains (VWC and CTCK) regulate the potential signaling pathways. However, after detecting the expression of key factors among Wnt/β-catenin, EMT, PI3K, and the mitogen-activated protein kinase (MAPK) pathways, we failed to observe any significant expression changes in the presence of the VWC and CTCK domains of MUC19 protein (Fig. S4a through d). Then, a recent CNV analysis identifies MUC19 as a highly mutated gene in hepatoid adenocarcinoma of the stomach (HAS) and a promising novel diagnostic marker for HAS (38). They found that overexpression of MUC19 leads to activated Wnt signaling exemplified by Wnt factors cyclin D1 (encoded by *CCND1*) and c-Myc. Therefore, we performed CRISPR-mediated knockout of MUC19 in Daudi cells and demonstrated that MUC19 modulates cyclin D1 expression but not c-Myc (Fig. 4a and b), indicating that MUC19 may utilize signaling pathways other than canonical Wnt signaling. Notably, cyclin D1 expression has been previously reported to depend on mTORC1 signaling (39, 40), implying a potential connection between MUC19 and mTORC1-regulated processes.

To further explore the role of MUC19 in EBV latency, we noticed the repeat region in the MUC19 protein, which contains tandem copies of “GVTGTTGPSA” (Fig. 3a). Motif analysis revealed approximately 351 copies of this basic repeat unit in MUC19 DNA (5′-GGAGTGACAGGGACAACTGGACCATCAGCT-3′, referred to as Re) (Fig. 4c). Of these, 338 copies are located within the EBV-induced duplicated CNV regions. To validate the biological functions of Re, we first overexpressed Re in HEK293T cells and found it also localized in the cytoplasm (Fig. 4d), which is similar to MUC19’s localization in B cells (Fig. 3b). Following the confirmation of Re expression (Fig. S4e), it was observed that Re overexpression upregulated several downstream factors of the mechanistic target of rapamycin (mTOR) signaling pathway, which further supports that Re can activate mTOR signaling (Fig. 4e and f). MUC19 knockdown in EBV-positive LCL1 cells leads to suppressed phosphorylation of mTOR and pan-PI3K, as well as cyclin D1 expression, demonstrating the critical role of MUC19 in mTOR signaling (Fig. 4g). Furthermore, Re overexpression in both HEK293T and Akata cells confirmed its role in mTOR activation through enhancing the phosphorylation of mTOR and pan-PI3K and increasing cyclin D1 expression (Fig. 4h). The mRNA expression of mTOR downstream factors *RPS6KB1*, *CCND1*, and *SGK1* was elevated following the increase of Re copies (Fig. 4i through k). Moreover, Re expression was found to elevate cell viability, underscoring its significance in promoting cell cycle progression and cell survival (Fig. 4l), suggesting its pro-survival role, which may have further oncogenic implications (40, 41). Therefore, these results indicate that the tandemly linked repeats within MUC19 have overlaying effects on mTOR activation, supporting its pro-survival role following partial duplication from EBV infection.

### EBNA1 binds to the MUC19 gene and enhances its expression

**Fig 5:**
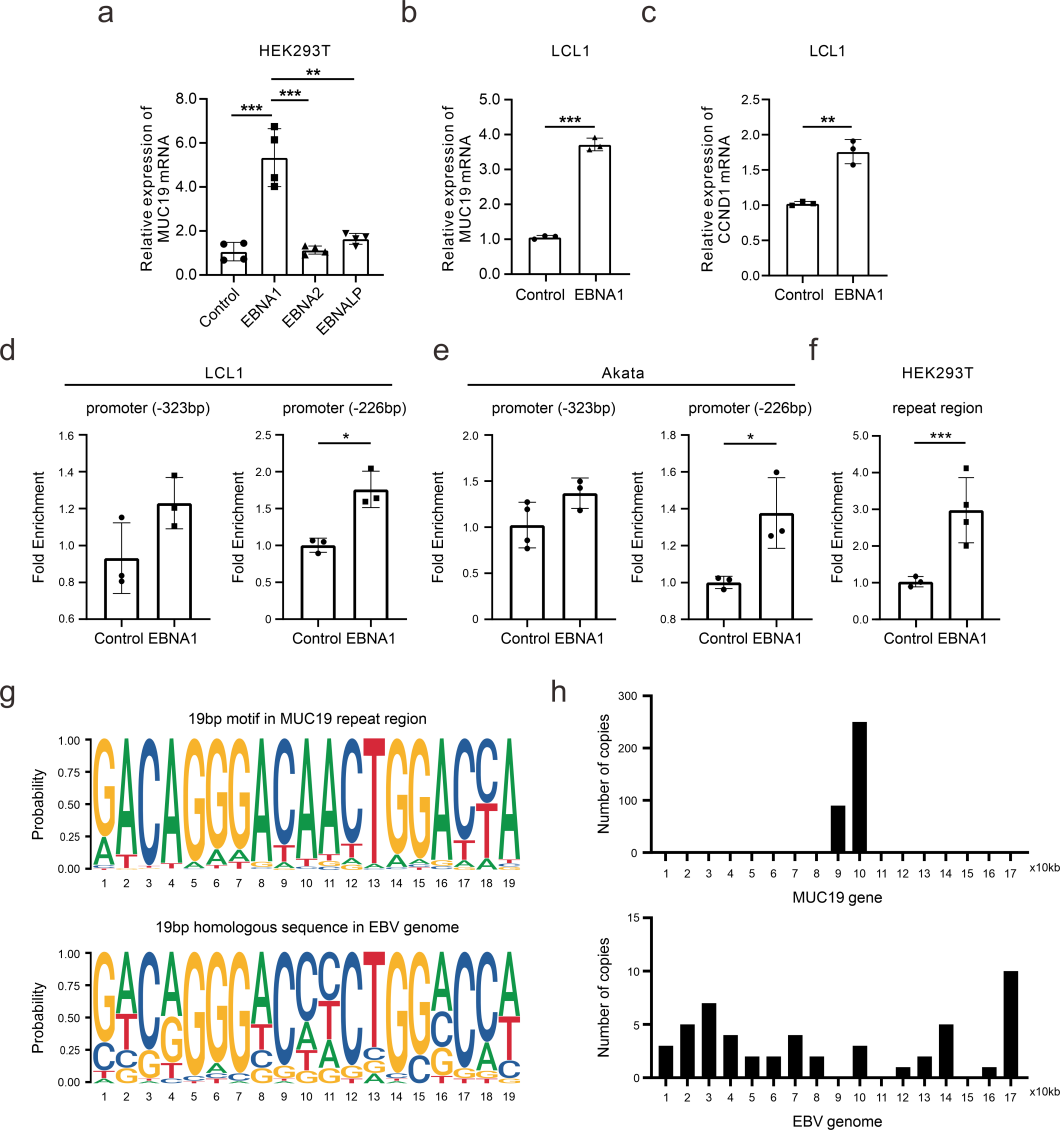
EBNA1 binds to the MUC19 gene and enhances its expression. (**a**) MUC19 mRNA expression was detected using qPCR after transient transfections of EBV nuclear antigens in HEK293T cells. (**b**) MUC19 mRNA expression was determined after transient EBNA1 transfection in LCL1 cells. (**c**) CCND1 expression was tested after transient EBNA1 transfection in LCL1 cells. (**d and e**) ChIP assays were utilized to detect the interaction between EBNA1 and the promoter region of *MUC19* in LCL1 (**d**) or Akata (**e**). (**f**) ChIP assay was used to verify the interaction between EBNA1 and the repeat region of MUC19. *, *P* < 0.05; **, *P* < 0.01; ***, *P* < 0.001. (**g**) A multiple alignment tool was used to discover a 19 bp motif in the MUC19 repeat region (5′-GACAGGGACAACTGGACCA-3′), which seems to be homologous to EBV intrinsic repeat. FIMO was used to identify the motif using a *P* value threshold of 0.00002. (**h**) The enrichment of the 19 bp motif in MUC19 DNA is shown in the density plot. The motifs identified are enriched in the CNV region of MUC19, while the corresponding 19 bp motifs in the EBV genome were dispersed throughout.

EBV nuclear antigens are shown to disrupt host genomic stability, potentially leading to oncogenic mutations (42). Among them, EBNA1 is a multi-functional EBV-encoded latent protein that is important for maintenance of the genome and also serves as a transcription activator (18, 43). EBNA2 and EBNALP are also EBV-encoded transcription factors (44). Overexpression of these antigens revealed that EBNA1 significantly activates MUC19 expression (Fig. 5a), which is also validated in EBV-positive LCL1 cells (Fig. 5b). EBNA1 can activate cyclin D1 expression that may be associated with MUC19 (Fig. 5c). To explore whether EBNA1 regulates MUC19 expression on transcriptional initiation, we performed chromatin immunoprecipitation (ChIP) assays in EBV-positive LCL1 and Akata cells with overexpressed EBNA1 and demonstrated that EBNA1 can bind to the MUC19 promoter region and facilitate its transcription (Fig. 5d and e).

EBNA1 is shown to aggregate around chromosomal regions and trigger double-strand breakage (19). Additionally, the ChIP assay revealed that EBNA1 actively interacts with the MUC19 repeat region (Fig. 5f). Together, these findings imply that EBNA1 may induce MUC19 CNVs in EBV infection. Furthermore, multiple sequence alignment indicates homology between the MUC19 repeat unit and the EBV genome (Fig. 5g). This homologous sequence specifically entailed 19 bp of the total 30 bp sequence and is the same as the intrinsic repeats among the EBV genome, such as BNRF1, EBNAs, BHLF1, BMRF1, and LF3 genes. In the MUC19 DNA sequence, 351 motif occurrences were identified within the MUC19 gene, while 51 homologous occurrences were found in the EBV genome (Fig. 5h). This interesting finding suggests homologous recombination of EBV with the host genome, specifically MUC19, during EBV infection.

**Fig 6:**
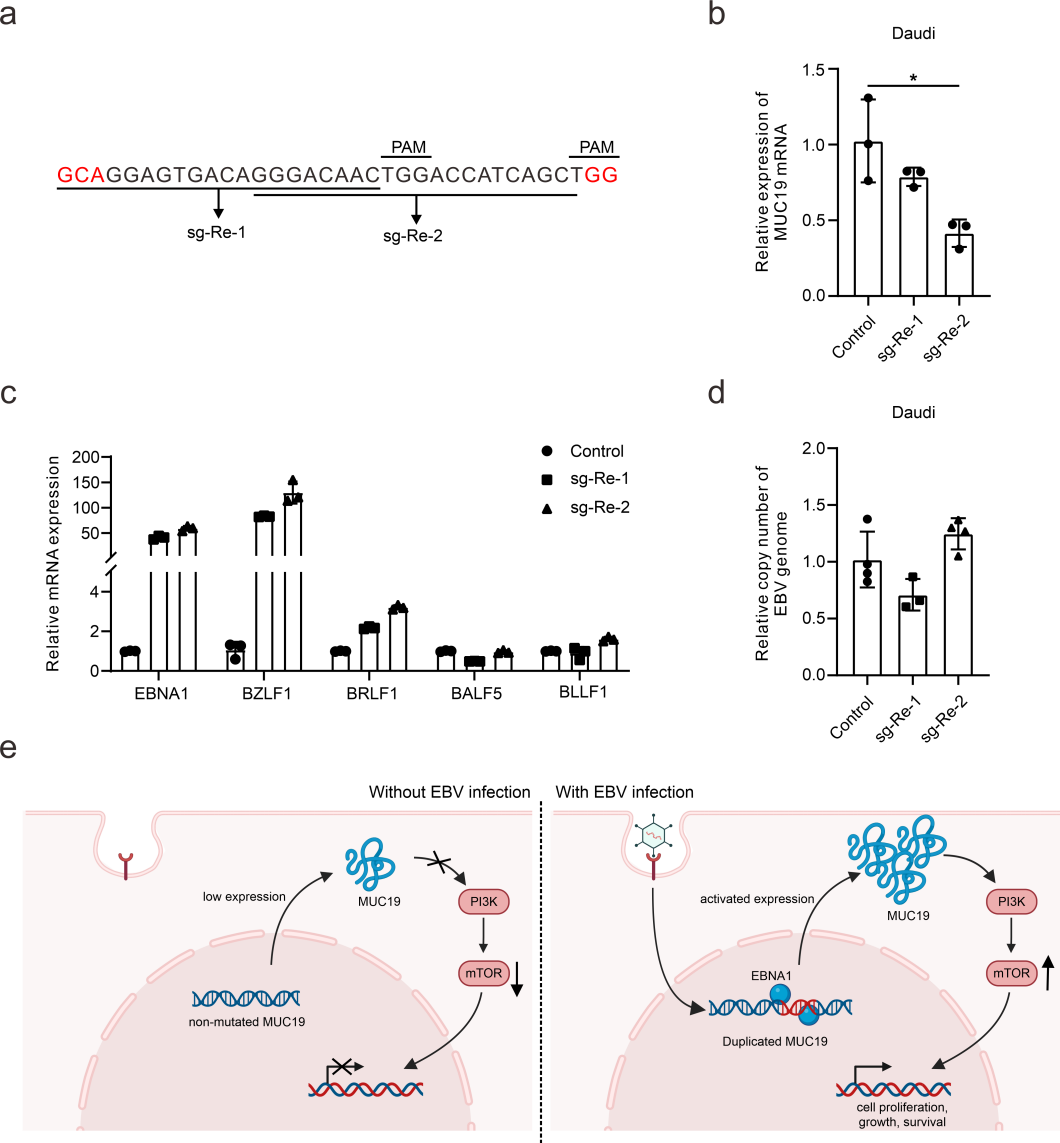
MUC19 tandem repeats are essential for inhibiting the initiation of EBV reactivation. (**a**) Two sgRNAs targeting the 30 bp MUC19 repeat unit were designed. PAM, protospacer adjacent motif helps Cas9 to recognize and bind target DNA, and the red nucleotides are the adjacent nucleotides outside the 30 bp repeat. (**b**) The Re region of the MUC19 gene was targeted with the CRISPR/Cas9 knockout system in Daudi cells. Then MUC19 mRNA expression was investigated with qPCR after targeting the 30 bp repeats. *, *P* < 0.05. (**c**) The expression of these indicated viral genes was detected following Re inhibition in Daudi cells. *EBNA1* is a viral latent gene; *BZLF1* and *BRLF1* are viral immediate-early genes; *BALF5* and *BLLF1* are viral late genes. (**d**) The EBV copy numbers were detected with qPCR after knocking out the MUC19 repeat region in Daudi cells. (**e**) The graphical abstract shows the model proposed by this study, which was created with BioRender (biorender.com).

Finally, to explore the functions of the MUC19 repeat unit on EBV dynamics, we developed sgRNAs targeting the indicated 30 bp repeat unit in EBV-positive Daudi cells (Fig. 6a). Disruption of the MUC19 repeats results in reduced MUC19 expression (Fig. 6b). Interestingly, disruption of the 30 bp repeat unit induces intense expression of viral immediate-early genes *BZLF1* and *BRLF1*, but not viral early gene (*BALF5*) or late gene (*BLLF1*) (Fig. 6c). Similarly, targeting the 30 bp repeat unit does not result in increased EBV replication or progeny virus production (Fig. 6d). These findings collectively indicate that MUC19 can inhibit the initiation of EBV reactivation. The tandem repeats of MUC19 are essential for this function, offering novel perspectives for developing targeted therapies against persistent EBV infection.

To conclude, we demonstrate that EBV infection promotes genomic instability via EBNA1, which triggers duplication within MUC19’s repeat region and activates its expression. The expressed MUC19 then activates mTOR signaling, thus promoting cell survival and cell cycle, finally contributing to EBV latency and EBV-mediated diseases (Fig. 6e).

## DISCUSSION

This study explored EBV-induced genomic perturbations in B cells on a copy number level and identified a specific CNV duplication of MUC19 in EBV-positive cell lines, indicating the critical role of MUC19 in EBV-mediated pathogenesis. These results showed that MUC19 expression is upregulated by EBV infection and promotes cell cycle through modulating the mTOR-cyclin D1 signaling pathway via its tandem repeats. Then, we confirmed that EBNA1 can bind to the promoter of the MUC19 gene and enhance its expression in B lymphoma cells. Finally, we showed that the MUC19 repeat region can inhibit the initiation of EBV reactivation.

Our study highlights the CNVs introduced by EBV infection throughout the human genome. Besides MUC19, there are other interesting CNVs shown from our primary infection model, including IRF4/DUSP22 duplication and Notch homolog 2 N-terminal-like protein A (NOTCH2NLA) deletion (Table S1; Fig. S5a and b). Interestingly, IRF4 and dual specificity phosphatase 22 (DUSP22) were identified to be within the same EBV super enhancer (45). These results indicate that DUSP22 was activated in EBV-positive B cells, suggesting its potential role in promoting EBV latency (Fig. S5a). NOTCH2NLA was downregulated in LCLs (Fig. S5b), possibly due to EBV-induced copy number deletion. However, NOTCH2NLA activates the Notch signaling pathway, which further contributes to EBV oncogenesis (46, 47). This change in expression brought by CNV cannot provide a functional explanation for its role in EBV latency through Notch signaling. However, it might still be important from angles other than Notch activation. Further experiments are required to further explore the role of NOTCH2NLA in EBV-related malignancies.

Furthermore, our findings suggest an EBV-specific MUC19 at its C-terminus, which could link to maintenance of EBV episome during latency. However, overexpression of its C-terminus fails to impact key pro-survival signaling pathways (Fig. S4a through d). Nevertheless, the C-terminus of MUC19 could have functional roles but requires further investigation. Although MUC19 is the only mucin factor identified from our screen with copy number duplication, mucin factors in general were differentially regulated upon EBV infection (Fig. 2g). For instance, the membrane-tethered MUC12 exhibited a similar expression pattern to MUC19 after EBV primary infection. The oncogenic role of MUC12 in renal cell carcinoma is related to the c-Jun/TGF-β signaling pathway (48). As the mucin family is tightly associated with numerous inflammatory pathways and immune responses in various pathologies, these genes may also be involved in EBV-associated biological processes. Future efforts may explore the roles of other mucin factors in regulating EBV-mediated diseases.

EBV primary infection has demonstrated that the observed genomic variations are EBV specific and identified EBNA1 as the underlying contributor, but the precise mechanisms have yet to be fully elucidated. We observed that EBNA1 overexpression in EBV-associated cells facilitates its binding to multiple regions of MUC19; thus, further investigation may be needed to determine whether the endogenous expression of EBNA1 operates similarly. Further studies could employ fluorescence staining techniques to observe interactions between viral proteins and specific genomic regions, as well as chromosomal breakages in adjacent regions, to confirm the occurrence of double-strand breaks (DSBs) and DNA repair. Additionally, we can explore the mechanisms underlying mutations during transcription. Previous studies found that the CHAMP1 complex promotes the assembly of heterochromatin at multiple chromosomal loci, including centromeres and telomeres, and facilitates homologous recombination repair of DSBs in these regions (49). Moreover, the CHAMP1 complex plays a crucial role in heterochromatin assembly and DSB repair in highly specialized chromosomal regions. This study suggests that DSBs may originate from secondary structures formed by single-stranded DNA and its binding to heterochromatin. The studies of heterochromatin organization in EBV primary infection could enhance our comprehension of EBV-host interactions on chromosomal modifications.

In conclusion, our study combined WGS data from EBV primary infection and other LCLs to characterize EBV-specific copy number variations. Integrative analysis and expression assays identified MUC19 as a critical factor for EBV infection. These findings unveil a novel role for MUC19 in regulating cell cycle through mTOR activation to promote EBV latency. Furthermore, we revealed that the repeat region is essential for MUC19-mediated mTOR activation and is closely connected with the EBV-encoded EBNA1. Therefore, the study elucidates genomic mutations that are induced by EBV primary infection and highlights the potential of MUC19 as a novel therapeutic target for EBV-associated malignancies.

## MATERIALS AND METHODS

### Plasmids

EBV latent genes (EBNA1, EBNA2, and EBNALP) are cloned into the pEGFP-C1 vector (Takara, Japan). The VC plasmid in the study was generated by cloning the VWC and CTCK domains (NM_173600.2: 24,529–25,205) into the pCMV-Myc-N vector (Takara) that was digested by EcoRI and XhoI. The Re plasmid was generated by cloning the basic repeat unit of MUC19 (5′-GGAGTGACAGGGACAACTGGACCATCAGCT-3′) into the pCMV-Myc-N vector (Takara), digested by EcoRI and XhoI. pMD2.G (Addgene #12259) and PsPAX2 (Addgene #12260) are used for lentivirus packaging. For CRISPR systems, the plasmid dCas9-KRAB-MeCP2-green fluorescent protein (GFP) is modified from lenti_dCas9-KRAB-MeCP2 (Addgene #122205) by replacing the BSD with GFP, which is a generous gift from Dr. Ruilin Tian (Southern University of Science and Technology, China). The sgRNA backbone plasmid pLG15-NC is also a gift from Dr. Ruilin Tian. Transient knockout using CRISPR/Cas9 is performed using lentiCRISPRv2 (Addgene #98290). sgRNA primers used for CRISPR systems are designed using the CHOPCHOP online tool (chopchop.cbu.uib.no/) and are listed in Table S2.

### Cells and antibodies

HEK293T (human embryonic kidney cell line) cells were cultured in Dulbecco’s modified Eagle’s medium supplemented with 10% fetal bovine serum (FBS), 50 U/mL penicillin, 50 µg/mL streptomycin, and 2 mM L-glutamine. EBV-negative cells (total-B, BL41, BJAB, DG75, and Ramos) and EBV-positive cells (EBVinf, Akata, Daudi, GM12878, LCL1, LCL2, Namalwa, and Raji) were cultured in RPMI 1640 medium supplemented with 10% fetal bovine serum, 50 U/mL penicillin, 50 µg/mL streptomycin, and 2 mM L-glutamine. All the cells were cultured in a 37°C incubator at 5% CO_2_. Transfections of HEK293T were performed using a Calcium Phosphate Cell Transfection Kit (C0508; Beyotime, China), and transfections into LCL1 and Akata were conducted using GenePulser Xcell Electroporation Systems (Bio-Rad, USA).

MUC19 antibody was purchased from R&D Systems (MAB8245). β-Actin and cyclin D1 antibodies were obtained from Proteintech (66009-1-Ig and 60186-1-Ig). pho-PI3K P85α/β/P55γ antibody was purchased from Beyotime (AF5905). mTOR, pho-mTOR, and PI3K p85α antibodies were obtained from Abmart (T55306, T56571, and T40115).

### EBV primary infection and whole genome sequencing

Approximately 10 million primary B cells were infected with EBV (B95.8, multiplicity of infection = 1) for 14 days, while 5 million B cells were stored at −140°C as a negative control. Then, both the infected and uninfected B cells were collected, and the genomic DNA was extracted from these obtained cells to establish a library using Nextera DNA Flex Library Preparation kits (Illumina, USA). Subsequently, the libraries were submitted to the Genome Technology Access Center at Washington University in St. Louis for whole genome sequencing using the NovaSeq 6000 system (Illumina). Besides collecting cells in EBV primary infection, other EBV-negative or EBV-positive cells were also sent for whole genome sequencing with the same procedures.

### Multi-omics sequencing data analysis

CNVkit (v.0.9.4) (50) was used to call CNVs from WGS data. The WGS data for primary B cells were used as a reference for the CNV analysis of the EBV-transformed cells. A comparative analysis was performed using bedtools (v.2.30.0) and R scripts. Visualizations were made using the R package circlize (v.0.4.16). The WGS data underwent standard processes for quality control and alignment to human genome assembly Hg38.

Somatic SNVs and small insertions and deletions were performed using GATK (51). Joint analysis of CNV and SNV was done by intersecting SNV loci with CNV segments using the R package GenomicRanges (v.1.52.0). The CNV regions were sorted in descending order based on the number of SNVs contained within each region. To normalize the data, mutation scores were calculated by dividing the number of SNVs and normalized by the length of the corresponding CNV region. The top 100 CNV segments with the highest mutation scores were selected for enrichment analysis.

RNA-seq data were analyzed using the sequencing results from EBV-infected primary B cells on days 0 and 14, which are available at the Gene Expression Omnibus (GEO) under accession number GSE125974 (28). The raw data of gene expression were quantified using Salmon (v.0.8.2). Differentially expressed genes were obtained using ESeq2 (v.1.40.2) in R, with a *P* value of <0.05 and absolute fold change of >5. Kyoto Encyclopedia of Genes and Genomes analysis was carried out using the R package clusterProfiler (v.4.8.3) and visualized by ggplot2 (v.3.5.1). ChIP-seq visualizations were performed using WashU Epigenome Browser (52). The identified Hi-C loops from GM12878 cells were acquired from GEO under data set GSE63525 (23).

### CRISPR systems

For the CRISPRi system, the dCas9-KRAB-MeCP2-GFP plasmid was transfected into Namalwa cells to establish stable cell lines through GFP fluorescence-activated cell sorting. Subsequently, sgRNAs targeting promoter regions of interest were designed using the CHOPCHOP online tool (chopchop.cbu.uib.no/) and cloned onto the pLG15-NC vector. These sgRNA constructs were then transfected into target cells, followed by puromycin selection at 2 µg/mL for 3 days and subsequent cell culture in the normal medium.

For the CRISPR/Cas9 system, the lentiCRISPR (v.2) plasmid was transfected using electroporation in both LCL1 and Daudi cell lines. LCL1 cells then underwent puromycin selection at 0.5 µg/mL for 3 days, followed by cell culture with 20% FBS, while Daudi cells underwent puromycin selection at 3 µg/mL for 3 days before cell culture with 20% FBS.

### Quantitative real-time PCR

Quantitative real-time polymerase chain reaction (qRT-PCR) was utilized to measure the expression of target genes. Total RNA was extracted using an RNA extraction kit (Beyotime). Samples with high-quality RNA (A260/A280 ratio between 1.8 and 2.0) were used for cDNA synthesis. The synthesized cDNA was used as a template for PCR amplification using gene-specific primers with SYBR Green Master Mix (Yeason, China). The qRT-PCR reactions were run on QuantStudio (v.5; Thermo Fisher Scientific, USA) with the following cycling conditions: initial denaturation at 95°C for 5 minutes, followed by 40 cycles of denaturation at 95°C for 15 seconds, annealing at 55°C for 30 seconds, and extension at 72°C for 30 seconds. The relative expression levels of the target genes were calculated using the 2^(−ΔΔCt)^ method. The qPCR primers designed in this study are listed in Table S2.

### Chromatin immunoprecipitation assay

Chromatin immunoprecipitation is performed using a ChIP assay kit (Beyotime). Ten million cells were collected 48 h post-transfection and were cross-linked using formaldehyde to a final concentration of 1% (0.68 mL of 37%/25 mL media) for 10 minutes. The cross-linking was stopped using glycine solution with a final concentration of 125 mM. Next, the cells were collected using centrifugation (700 × *g*, 4 min) and lysed using ChIP lysis buffer. After sonication, the lysates were incubated with salmon sperm DNA/protein A agarose beads, together with 5 µg EBNA1 antibody or normal IgG antibody, and placed on a rotating platform overnight at 4°C. The next day, the formaldehyde cross-links were reversed, and the collected DNA was purified with a DNA purification kit (TIANGEN, China).

The primers used for targeting the promoter (−323 bp) were 5′-TCTGGGTTTGGTATGAGCTGG-3′ and 5′-GTCTGCCATCATGGGCTAGG-3′; those used for targeting the promoter (−226) were 5′-CACAGGGTGGCAAGAAGACA-3′ and 5′-TGGGCTAGGTGTGGTAAACT-3′. The primers used for the repeat region were 5′-GGACAACTGGACCATCAGCT-3′ and 5′-TCCAGTTGTCCCTGTCACTCC-3′.

### Cell viability assay

Cell viability was measured using the Cell Counting Kit-8 (Beyotime). Briefly, the indicated cells were transferred to a 96-well plate at a density of 5,000 cells per well in a 100 µL medium. Ten microliters of CCK-8 solution was then added to each well. Then, the optical density at 450 nm was measured 2 h after the addition of the CCK-8 reagent using BioTek Synergy H1 microplate reader (Agilent Technologies, USA).

### Immunofluorescence

The B cells are centrifuged and air-dried on coverslips. Then they were fixed with 4% paraformaldehyde, permeabilized with 0.1% Triton X-100 for 10 minutes, and blocked with 5% bovine serum albumin (BSA) for 20 minutes at room temperature. Primary antibodies against the target protein were diluted at a ratio of 1:1,000 using phosphate-buffered saline (PBS)-B (4% BSA) and incubated overnight at 4°C. After washing with PBS, the cells were incubated with fluorochrome-conjugated secondary antibodies (1:1,000 diluted) for 1 h at room temperature in the dark. Nuclei were counterstained with 4′,6-diamidino-2-phenylindole. Fluorescent images were taken using a confocal microscope LSM 900 (Zeiss, Germany). Image processing was performed using Zen blue (v.3.4.91).

### Western blot and enzyme-linked immunosorbent assay

The cells were harvested and lysed for protein extraction. Then the proteins were resolved on SDS-PAGE gel and transferred to a polyvinylidene fluoride (PVDF) membrane. This membrane was blocked with 5% skim milk, followed by overnight incubation with a specific primary antibody. After washing the membranes, they were incubated with horseradish peroxidase (HRP)-labeled secondary antibody for 1 h, and the membrane was washed again. Finally, the signals were detected through chemiluminescence, and the intensity of protein bands was quantitatively measured using the image analysis software ImageJ (v.1.54). Additionally, the enzyme-linked immunosorbent assay (ELISA) was performed to evaluate the secretion of MUC19 in B lymphoma cells using a commercially available ELISA kit (CUSABIO, China).

### Flow cytometry assay

This assay was determined using the Cell Cycle and Apoptosis Analysis Kit (Beyotime), which employs the propidium iodide staining method for cell cycle and apoptosis analysis. After staining the cellular DNA with propidium iodide, flow cytometry was performed using FACSCanto SORP (BD Biosciences, USA), and the results were analyzed with FlowJo (v.10.6.2).

### Motif discovery

The motif discovery was conducted using FIMO (v.5.5.5) (53). The *P* value was defined as the probability of a random match for the desired sequence with as good or better a score. The score was computed by summing the appropriate entries from each column of the position-dependent scoring matrix that represents the motif. R package ggseqlogo (v.0.2) was used to visualize the motifs.

## Data Availability

The raw whole genome sequencing data supporting the findings of this study are available in the National Center for Biotechnology Information Sequence Read Archive under BioProject accession number PRJNA1213170.

## SUPPLEMENTAL MATERIAL

Figure S1 mbio.02055-25-s0001.tif EBV primary infection induces SNP modifications on host chromosomes.

Figure S2 mbio.02055-25-s0002.tif EBV infection-induced CNVs are further annotated to reveal associated gene expression within the corresponding CNV regions.

Figure S3 mbio.02055-25-s0003.tif The function of MUC19 is relatively conserved among the mucin family.

Figure S4 mbio.02055-25-s0004.tif MUC19 may be involved in the specific signaling pathways related to EBV-mediated oncogenesis.

Figure S5 mbio.02055-25-s0005.tif EBV-induced CNV deletions may cause differential expression in various B cells.

Legends mbio.02055-25-s0006.docx Legends for supplemental materials.

Table S1 mbio.02055-25-s0007.xlsx The identified CNVs induced by EBV primary infection.

Table S2 mbio.02055-25-s0008.xlsx The primers used in this study.

Table S3 mbio.02055-25-s0009.xlsx Source data.

ASM does not own the copyrights to Supplemental Material that may be linked to, or accessed through, an article. The authors have granted ASM a non-exclusive, world-wide license to publish the Supplemental Material files. Please contact the corresponding author directly for reuse.
