# Supplementary figures and images for: Genome-wide mapping of EBV-induced genomic variations identifies the role of MUC19 in EBV latency
Source: mBio. 2025 Sep 25;16(11):e02055-25. doi: 10.1128/mbio.02055-25 (PMC12607598; doi:10.1128/mbio.02055-25)

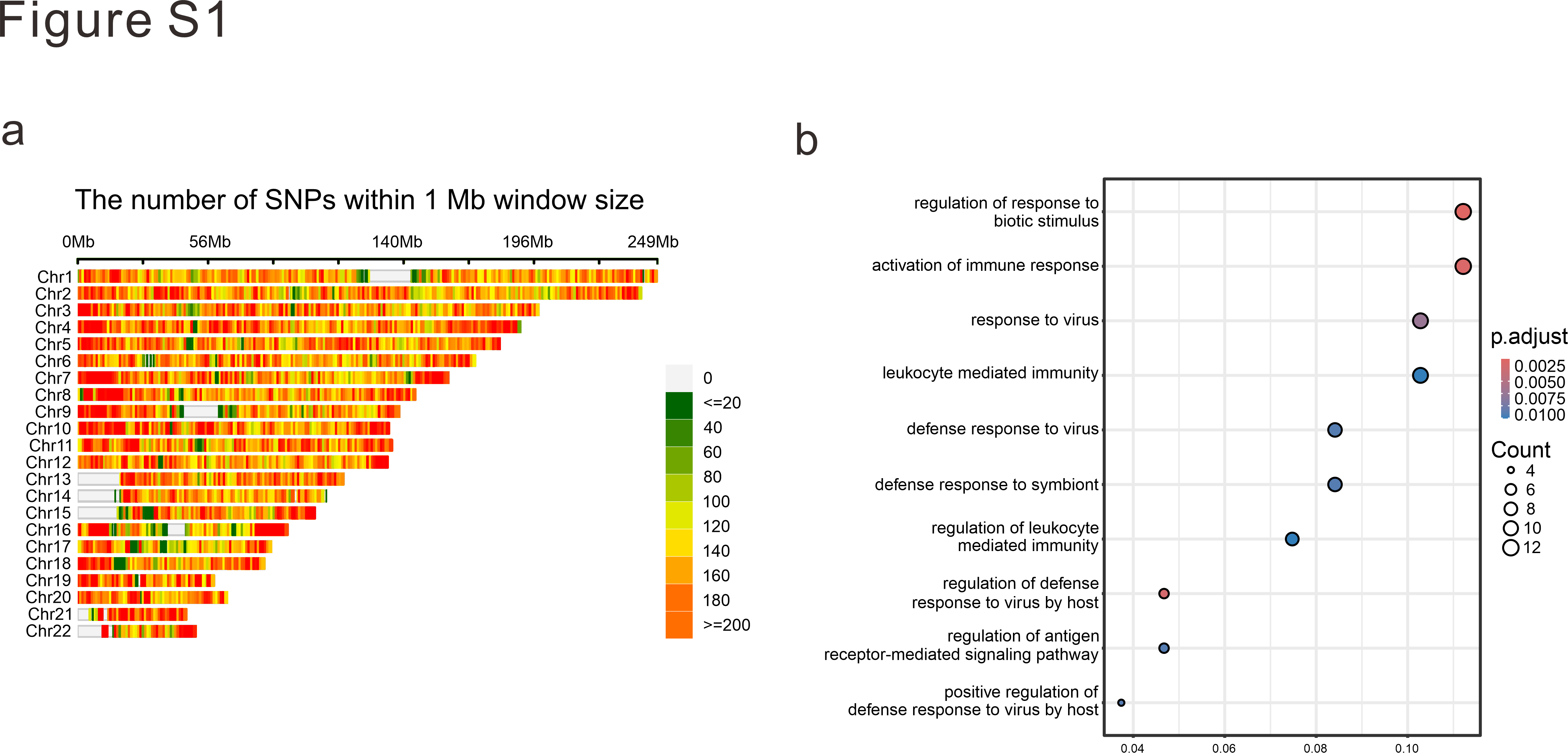

Supplement: Figure S1 — EBV primary infection induces SNP modifications on host chromosomes. [file mbio.02055-25-s0001.tif]

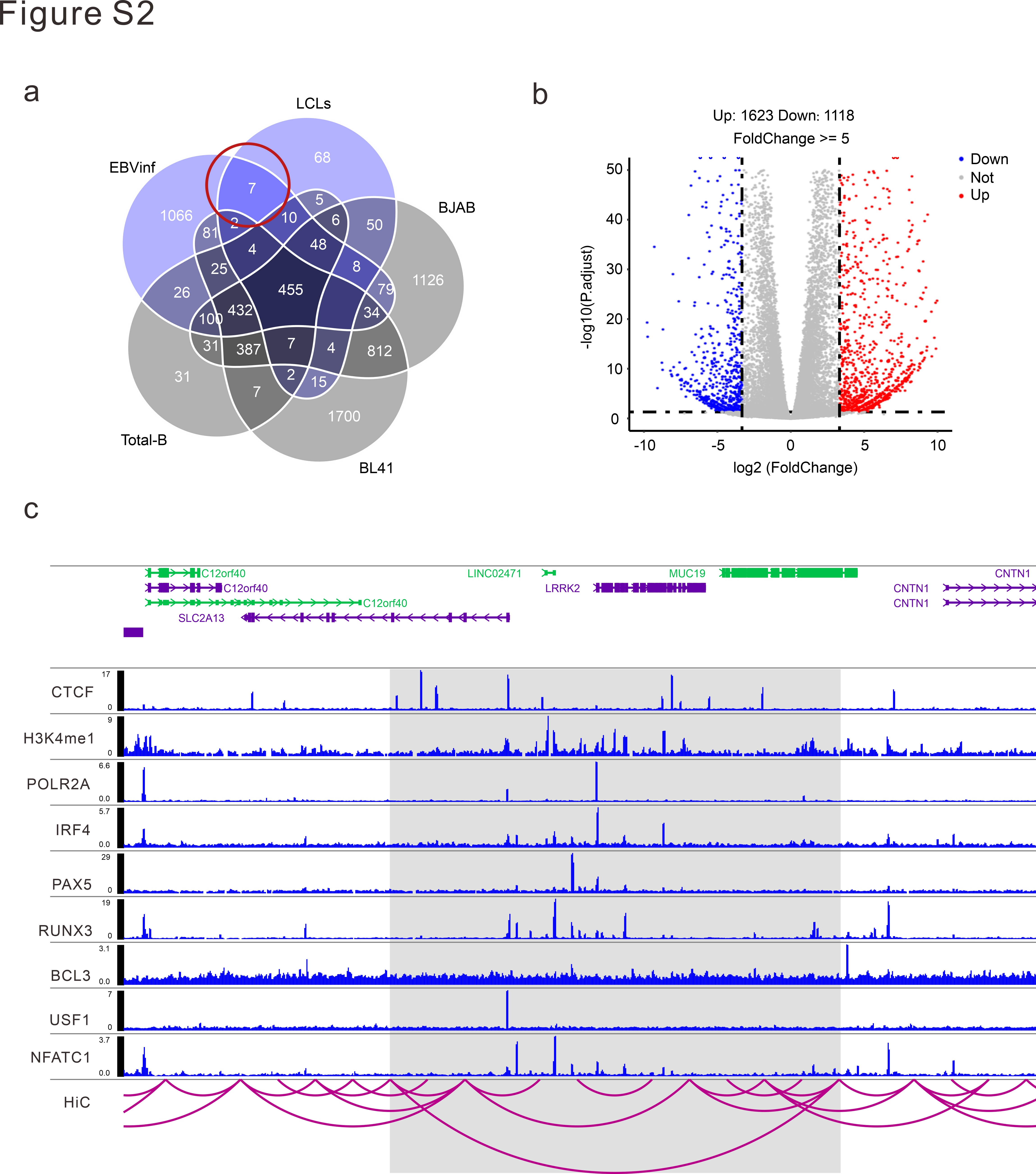

Supplement: Figure S2 — EBV infection-induced CNVs are further annotated to reveal associated gene expression within the corresponding CNV regions. [file mbio.02055-25-s0002.tif]

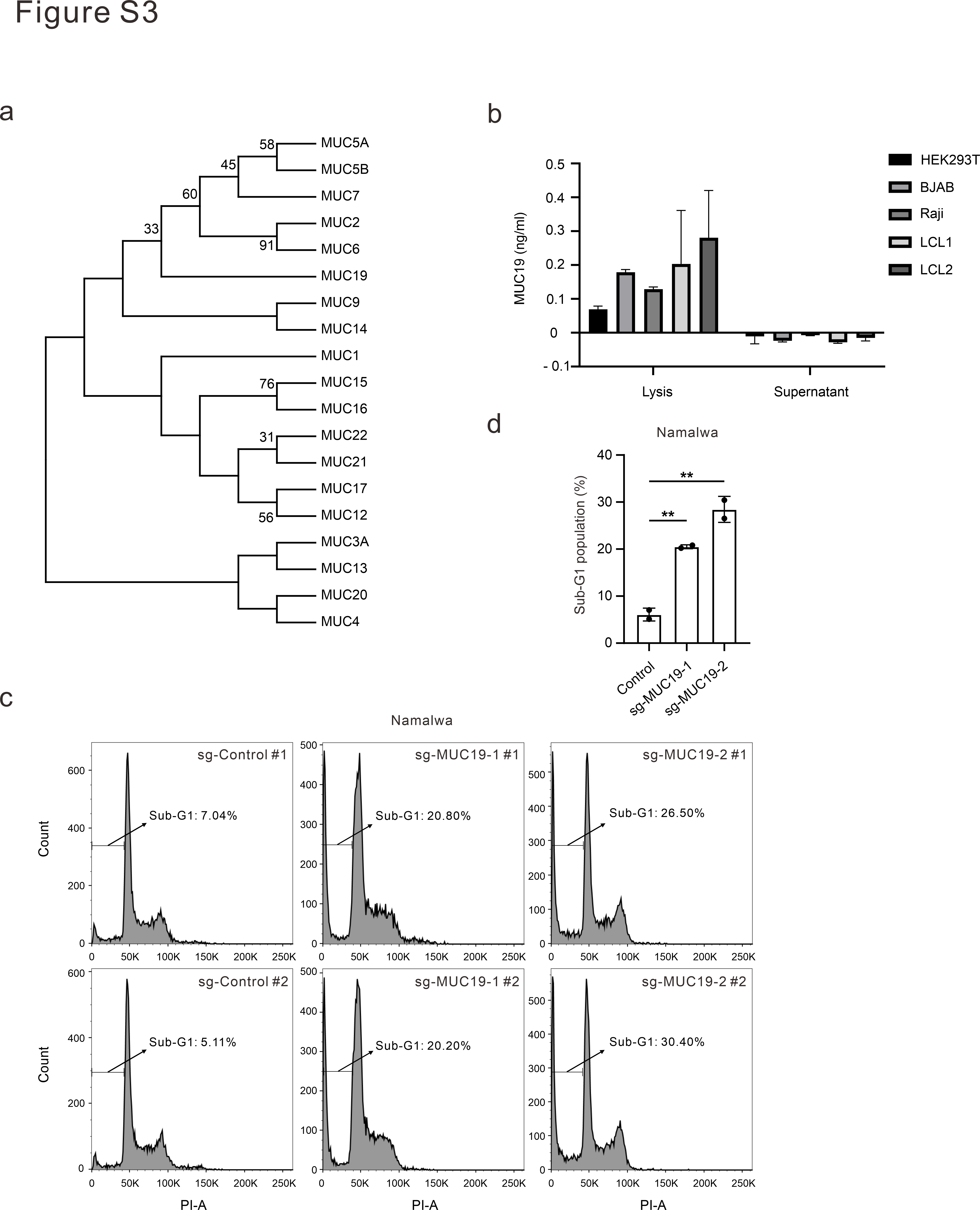

Supplement: Figure S3 — The function of MUC19 is relatively conserved among the mucin family. [file mbio.02055-25-s0003.tif]

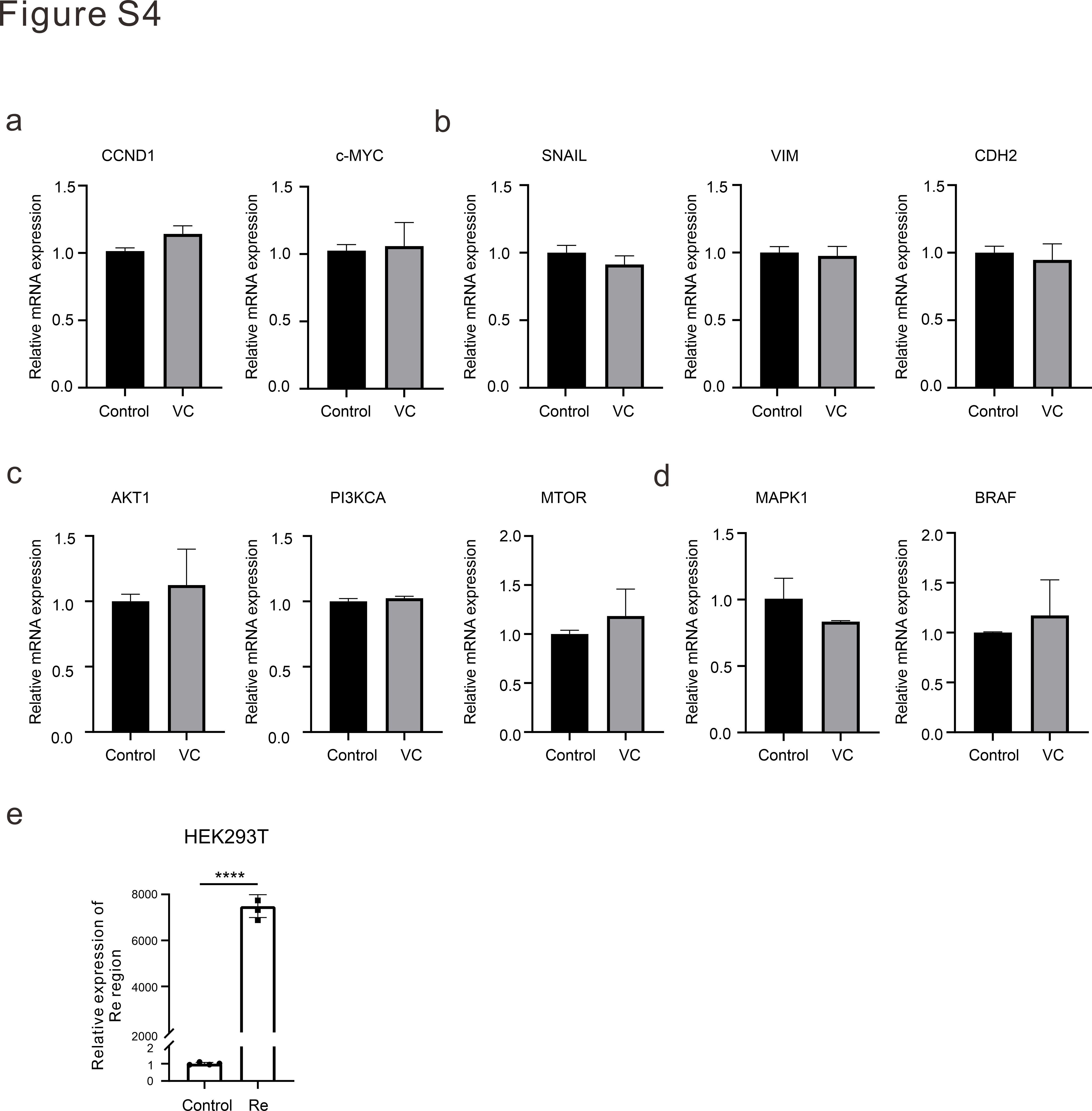

Supplement: Figure S4 — MUC19 may be involved in the specific signaling pathways related to EBV-mediated oncogenesis. [file mbio.02055-25-s0004.tif]

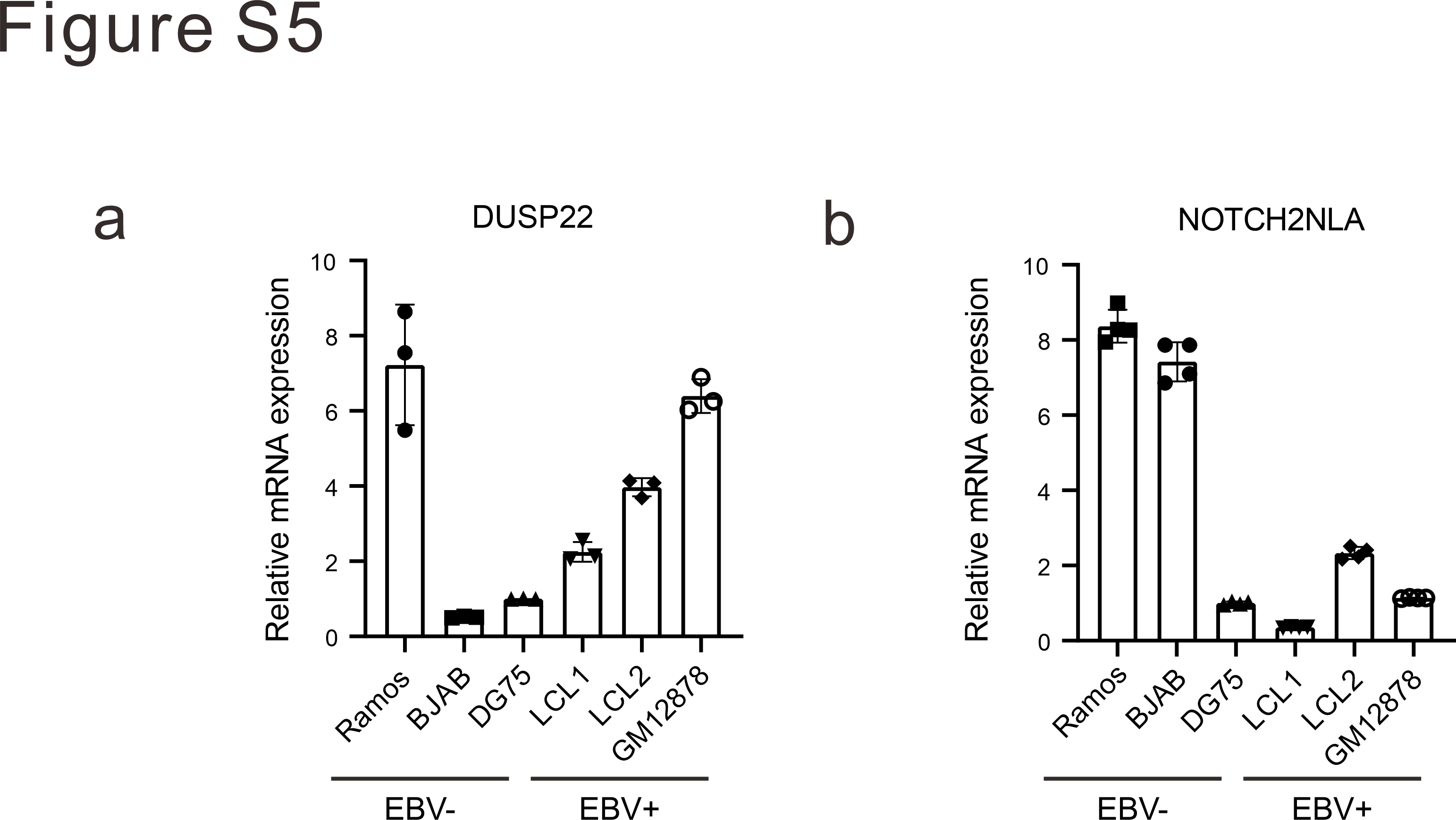

Supplement: Figure S5 — EBV-induced CNV deletions may cause differential expression in various B cells. [file mbio.02055-25-s0005.tif]
