# Supplementary material for: Genome-wide mapping of EBV-induced genomic variations identifies the role of MUC19 in EBV latency
Source: mBio. 2025 Sep 25;16(11):e02055-25. doi: 10.1128/mbio.02055-25 (PMC12607598; doi:10.1128/mbio.02055-25)
Supplement: Legends — for supplemental materials. [file mbio.02055-25-s0006.docx]

**Supplementary materials**

**Figure S1.** EBV primary infection induces SNP modifications on host chromosomes. (a) EBV introduces SNPs across the human genome. (b) The highly mutated regions are shown to possess anti-viral factors.

**Figure S2.** EBV infection-induced CNVs are annotated to reveal associated gene expression within the corresponding CNV regions. (a) Comparative analysis reveals genomic CNV deletions shared among EBV-related cell lines. (b) RNA-seq analysis identified the differentially expressed cellular factors during EBV primary infection. The samples on day 0 and day 14 were obtained from the GEO dataset GSE125974, with an absolute fold change threshold of 5, and a p-value threshold of 0.05. (c) The *LRRK2/MUC19* region (shaded) is highly regulated by EBV-related transcription factors.

**Figure S3.** The function of MUC19 is relatively conserved among the mucin family. (a) Comparative genomics shows the homology among all identified mucin factors. Bootstrap confidence greater than 30 is listed beside the branches. (b) The secretion of MUC19 among different B cells was detected using ELISA. Supernatants were collected after 48 hours of cell culture. (c-d) Flow cytometry was used to analyze cell cycle and apoptosis induced by MUC19 knockdown in Namalwa cells. The cells were fixed with 70% ethanol immediately after the establishment of stable cell lines.

**Figure S4.** MUC19 may be involved in the specific signaling pathways related to EBV-mediated oncogenesis. (a-d) The expression of key downstream factors for the (a) Wnt, (b) EMT, (c) PI3K/Akt, and (d) MAPK signaling pathways were detected in HEK293T cells with overexpressed MUC19 C-terminal domains. VC, the C-terminal VWC and CTCK domains of MUC19. (e) Re expression was determined with qPCR following transient transfection in HEK293T cells. ****, p < 0.0001.

**Figure S5.** EBV-induced CNV deletions may cause differential expression in various B cells. *DUSP22* is differentially expressed (a), while *NOTCH2NLA* is silenced (b) in EBV-related B cells.

**Table S1.** The identified CNVs induced by EBV primary infection.

**Table S2.** The primers used in this study.

**Table S3.** Source data.
